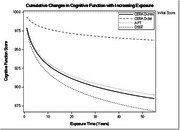# Dose‐Response Relationship Between Occupational Exhaust Fume Exposure and Cognitive Decline in Older Adults

**DOI:** 10.1002/alz70860_102480

**Published:** 2025-12-23

**Authors:** Xinyi Zhang, Lili Liang, Rose Saint Fleur‐Calixte, Perry E Sheffield, Tianxu Xia, Jenny J Lin

**Affiliations:** ^1^ Icahn School of Medicine at Mount Sinai, New York, NY, USA; ^2^ Teachers College, Columbia University, New York, NY, USA; ^3^ SUNY Downstate Medical Center, New York, NY, USA

## Abstract

**Background:**

Occupational exposure to diesel exhaust (DE) fumes has been linked to adverse health outcomes, including respiratory and cardiovascular diseases. However, its effects on cognitive function remain underexplored. This study investigated the association between occupational exhaust fume exposure duration and cognitive performance among older adults, highlighting a dose‐response relationship.

**Method:**

We utilized data from the 2011–2012 National Health and Nutrition Examination Survey (NHANES), focusing on adults aged 60 years and older (*n* = 1,110). Occupational exposure was assessed via self‐reported history of exposure to exhaust fumes, with exposure duration (OCQ560) used to examine cumulative effects. Cognitive performance was evaluated using the Consortium to Establish a Registry for Alzheimer's Disease Word List Learning Test (CERAD‐WL), the Animal Fluency Test (AFT), and the Digit Symbol Substitution Test (DSST). Weighted multivariable survey regression models adjusted for key confounders, including demographic and health‐related factors. Decline rates in cognitive scores were calculated to reflect yearly changes in performance.

**Result:**

Cognitive performance declined progressively with longer exposure to exhaust fumes. The dose‐response analysis revealed the steepest declines occurred within the first decade, particularly in processing speed (DSST: yearly decline = 3.5%), executive function (AFT: yearly decline = 2.9%), and delayed memory (CERAD‐WL Delay: yearly decline = 0.9%). Immediate memory (CERAD‐WL Immediate) demonstrated a slower decline (yearly decline = 0.3%). Over 50 years, DSST and AFT scores dropped by over 10%. The multivariable‐adjusted logistic regression model demonstrated significant associations between exposure and poor cognitive outcomes, with occupational exposure increasing the odds of impaired delayed memory (OR = 2.55, 95% CI: 1.61–4.05), verbal fluency (OR = 2.41, 95% CI: 1.48–3.94), and processing speed (OR = 1.95, 95% CI: 1.19–3.18).

**Conclusion:**

Occupational exposure to exhaust fumes is associated with accelerated cognitive decline, with a clear dose‐response relationship observed in key cognitive domains. Early declines emphasize the importance of timely interventions to mitigate long‐term impacts. Public health measures, such as enhanced workplace ventilation and transitioning to cleaner energy sources, may reduce occupational exposures and protect cognitive health. Future research should adopt longitudinal designs and explore the biological mechanisms underlying these associations and examine other environmental factors that may compound risks.